# Chemical Composition and Repellency of *Origanum vulgare* Essential Oil against *Cimex lectularius* under Laboratory Conditions

**Published:** 2018-12-25

**Authors:** Mona Sharififard, Ismaeil Alizadeh, Elham Jahanifard, Changlu Wang, Mohammad Ebrahim Azemi

**Affiliations:** 1 Infectious and Tropical Diseases Research Center, Health Research Institute, Ahvaz Jundishapur University of Medical Sciences, Ahvaz, Iran; 2 Department of Medical Entomology and Vector Control, School of Public Health, Ahvaz Jundishapur University of Medical Sciences, Ahvaz, Iran; 3 Department of Entomology, Rutgers University, New Brunswick, NJ, USA; 4 Department of Pharmacognosy, Medicinal Plant and Natural Product, Research Center, School of Pharmacy, Jundishapur University of Medical Sciences, Ahvaz, Iran

**Keywords:** *Cimex lectularius*, Essential oil, *Origanum vulgare*, repellency

## Abstract

**Background:**

The common bed bug (*Cimex lectularius*), a nocturnal blood-feeding ectoparasite, is considered an urban pest with public health importance in human environments. We aimed to determine the repellency effect of oregano essential oil, *Origanum vulgare*, against this pest under laboratory conditions.

**Methods:**

The essential oil was prepared from dried leaves using hydro-distillation method. A gas chromatography-mass spectrometer (GC-MS) was used for analysis and identification of oregano essential oil compounds. Treated surface method in Petri dish was carried out to evaluate repellency potential of the oil using 4th and 5th instar nymphs and adults. The concentration-repellency response of oregano essential oil was calculated and compared with a commercial insect repellent stick containing 33% N, N-diethyl-*meta*-toluamide (DEET).

**Results:**

The oregano essential oil consisted of 158 compounds with terpineol (22.85%) and α–terpinene (20.60%) being the major components by volume. The EC_50_ and EC_99_ of oregano oil (effective concentrations causing 50% and 90% repellency of bed bugs) were 1.61 and 6.57mg/cm^2^ at 9h after application, respectively. The 40% oregano essential oil exhibited 100% repellency against bed bug at 3, 5, 9 and 24h after application while the repellency index of DEET 33% was 100% at 3 and 5h and it decreased to 80% and 27% at 9 and 24 hours.

**Conclusion:**

Oregano oil 40% exhibited more repellency compared to commercial insect repellent stick containing 33% DEET. Further studies are warranted to confirm the effectiveness of oregano essential oil in personal protecting against bed bug biting.

## Introduction

Bed bug (*Cimex lectularius* Linnaeus), is a nocturnal blood-feeding ectoparasite considered as urban, common and nuisance pest in the recent past (1, 2). This insect affects everyone in different social status and infests everywhere. The case of infestation has been reported from countries including United Kingdom, Denmark, Norway, Italy, Spain, Sweden, Scandinavia, Switzerland, Australia, Brazil, Iran, Thailand, Malaysia, Singapore, Kuwait, Nigeria and the United States (2–11). The bed bug infestation became rare with the new pest control techniques in North America and Europe a century ago (12), but the United States has significant resurgence as a perfect storm in 2006 (13). The global resurgence of bed bug infestations was related to increased levels of international transport through tourism and trade, immigration insecticide resistance and possibly increased temperatures (14). Besides pain and itchiness, the problems caused by bed bug infestations include psychological distress with nightmares, insomnia, anxiety, social isolation, and quality of life (15).

Bed bugs hide in protected places such as crevices on beds and other furniture, inside devices, inside walls, floor cracks. This partially explains why they are difficult to detect and control (16). Surface treatment with pyrethroids and other classes of insecticides, physical control, and personal protection are the main recommendations for the control and prevention of bed bugs infestation (1, 17, 18). Application of insecticides poses an immediate risk to human health and the environment especially when insecticides are applied on furniture (17).

Personal protection using repellent compounds on human skin is an effective and in some cases the only practical approach to the control of biting insects. The most common synthetic repellent used on blood-sucking arthropods is N, N-diethyl-*meta*-toluamide (DEET) produced in 1954 (19, 20). Due to reports of allergic and toxic effects especially on children and pregnant women from DEET, it is placed under investigation (21–23). Therefore, finding a safer repellent compound for use on human body surface is necessary. Botanical essential oils are regarded as environmentally friendly products, ecological alternatives and low mammalian toxicity materials (24). However, some plant-based repellents should be used with caution due to their compounds (25).

Essential oils have been introduced as green pesticides and most of them are non-toxic to humans, animals and are safe and friendly to the environment (26). These products are good alternatives to synthetic insecticides and can delay the development of resistance to insect pests (27). Essential oils (EO) are secondary metabolites of plants. They are complex mixtures of volatile organic compounds such as hydrocarbons and oxygenates (28, 29). Today, many essential oil products have been developed and are effective as repellents or surface spraying compounds on pest of medical important (17, 30). Essential oils have various activities against insect pests, including insecticidal, antifeedant, repellent, oviposition, deterrent and growth regulatory (30, 31). Repellent activity of essential oils is an effective way of controlling biting insects including bed bugs and they can be applied on luggage, fabric materials and furniture in order to reduce bed bug infestation by preventing bed bugs into their home (27).

Oregano oil is considered as an excellent antiseptic and insect repellent. It has some active ingredients such as carvacrol, thymol and α-terpinene reported being highly effective in repelling mosquitoes (32, 33). Moreover, it showed significant repel activity at concentration range of 2.5–30% against *Supella longipalpa* (34).

The aim of this study was to determine the repellency activity of oregano essential oil (*O. vulgare*) on bed bugs in comparison with DEET against bed bugs (*C*. *lectularius*).

## Materials and Methods

### Bed Bugs

The adult and nymph stages of *C. lectularius* were collected from infested homes in Ahvaz City, southern Iran and transferred to the Laboratory of Medical Entomology Department, School of Public Health, Ahvaz, Jundishapur University of Medical Sciences, Ahvaz, Iran. The insects were reared in plastic containers (12cm in height and 6cm in diameter) with folded papers as harborage and kept at 26±1 °C, 50±5% relative humidity (RH), photoperiod of 12:12 (L: D) (17, 27). The colony of *C. lectularius* were fed weekly on rabbits and allowed to suck blood for 10min (2). The bed bugs were starved for 7 d before bioassays.

### Chemical compounds

Insect repellent stick (33% DEET) as commercial formulation was purchased from Reyhan Naghsh Jahan Pharmaceutical Cosmetic and Hygienic Company. It is used widely in Iran on blood-sucking insects such as mosquitoes, sand flies, ticks and bed bugs.

### Essential oil isolation

Oregano plant (*O. vulgare*) was collected from its natural habitat from Yazd Province, central part of Iran (latitudes 54.20257 and longitudes 32. 00315). Collected Oregano specimens were identified by the Department of Pharmacognosy, Ahvaz Jundishapur University of Medical Sciences. Firstly, fresh leaves dried with air, and next 200gr of oregano was mixed with 400ml of distilled water and placed in a 1L flask. In the following the essential oil oregano used in the assay was isolated from dried fresh leaves by the hydrodistillation method using a Clevenger-type apparatus (Model BP, British Pharmacopoeia, Manufacturer Pyrex Fan Company, Iran and mantle model H610 manufacturer Fater Company, Iran) at 90±5 for 5 hours. We extracted 0.9cc pure (about 100%) essential oil of 100gr of dried leaves of *O. vulgare*. Sodium sulfate was used for dehydration. The extracted essential oils were stored at 4 °C in dark glass vials for further experiment (35).

### Gas chromatographic-mass spectral analysis

Gas-chromatography-mass spectrometer (GC-MS) was used for the analysis and identification of oregano essential oil compounds (Hewlett-Packard 6890, Agilent Technology, Santa Clara, California, USA). It is equipped with HP–5MS column (30m× 0.25mm× 0.25 μm). The initial temperature used was 40 °C for 1min and was later raised to 220 °C at a rate of 3 °C/min and finally raised to 270 °C for 5min at a rate of 20 °C/min. Other parameters of the GC-MC machine included carrier gas Helium (99/999%), injector temperature (260 °C), detector temperature (FID, 270 °C), splitless mode, ionization potential of 70eV, scan rate of 1 scan/sec, the scan range of m/z 40–48 was used for all analysis. The essential oil constituents were identified by comparing their retention indices, mass spectra fragmentation with those in a stored Wiley 7n.1 mass computer library and those of National Institute of Standards and Technology (NIST) (36).

### Petri dish repellency assay

**Fig. 1.**
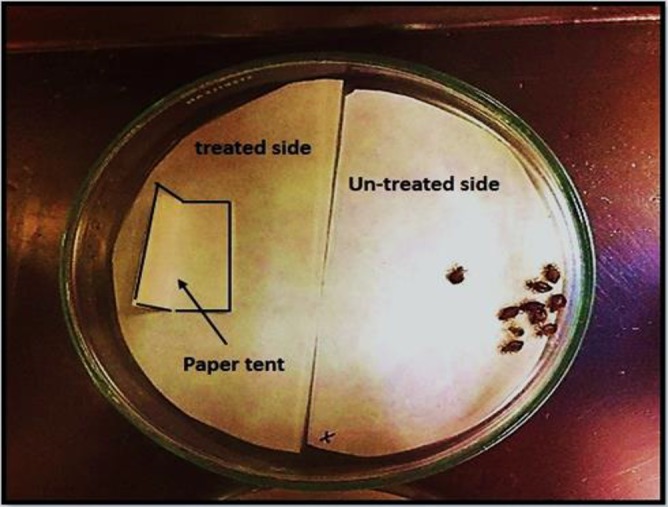
Petri dish repellency assay of oregano essential oil, ethanol and 33% DEET on bed bugs

Oregano essential oil at concentrations of 0.625, 1.25, 2.5, 5, 10, 20 and 40% (V/V) were evaluated against the laboratory-reared bed bugs in plastic Petri dishes and compared with DEET (33%). Moreover, the concentrations were calculated as doses of 0.1, 0.21, 0.43, 0.86, 1.72, 3.45, 6.9mg/cm^2^ for drawing dose-response curve. Plastic Petri dishes of 8cm diameter by 1.5cm height were used in this experiment. Ethanol was used as solvent for essential oil. White filter papers were divided into two equal halves and inserted into the bottom part of the Petri dishes. A piece of folded filter paper was placed in the middle as bed bug harborage (Fig. 1). This part (bed bug harborage) was treated with 0.7ml of essential oil solution using a micro-sampler and the other half was left untreated (27). In the control (negative and positive) plastic Petri dishes group, one half of the filter paper and the harborage were treated with 0.7ml of 96% ethanol or 33% DEET, and the other half was left untreated. Ten-bed bugs (4^th^ and 5^th^ instars, nymphs and adults) for each treatment group were released into the center of each Petri dish (Fig. 1). The number of bed bugs on each side of the Petri dish was recorded at 3, 5, 9, and 24h post treatment by visual inspection. The experiments were replicated four times for any concentrations in oregano essential oil, Ethanol, and 33% DEET. All Petri dishes were kept at 26±2 °C, photoperiod of 12:12 (L: D) and 55±5% relative humidity (RH).

### Statistical Analysis

Probit analysis was used to calculate the effective concentration (EC_50_ and EC_99_). Repellency indices were calculated using: repellency =(C–T)/C×100, where C is the mean number of bed bugs located on the treated filter paper in all control plastic Petri dishes, and T is the number of bed bugs located in half part of the treated filter paper at unfixed concentration test in the plastic Petri dishes (27). Repellency indices were compared using analysis of (ANOVA) followed by Dunnett test to distinguish between the treatments. All statistical analyses were performed using SPSS ver. 16 (Chicago, IL, USA).

## Results

### Yields and chemical constituents of essential oil

**Fig. 2.**
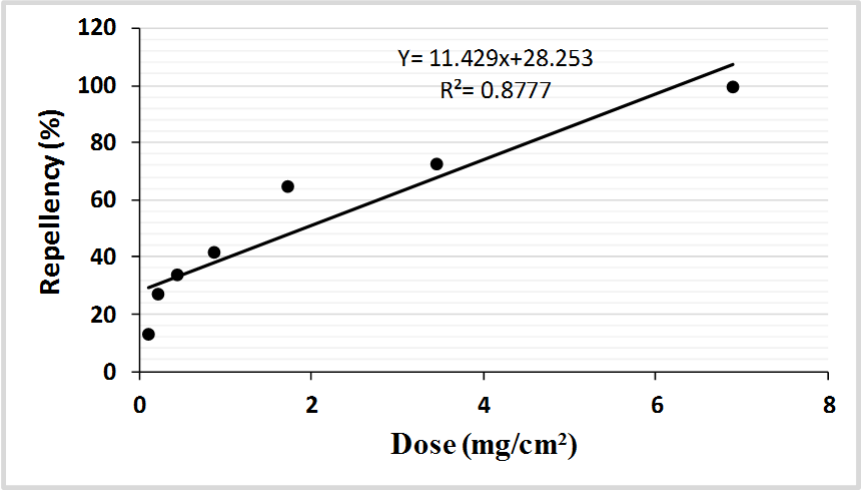
Dose-response curve for Oregano essential oil after 9 hour in laboratory assay

**Fig. 3.**
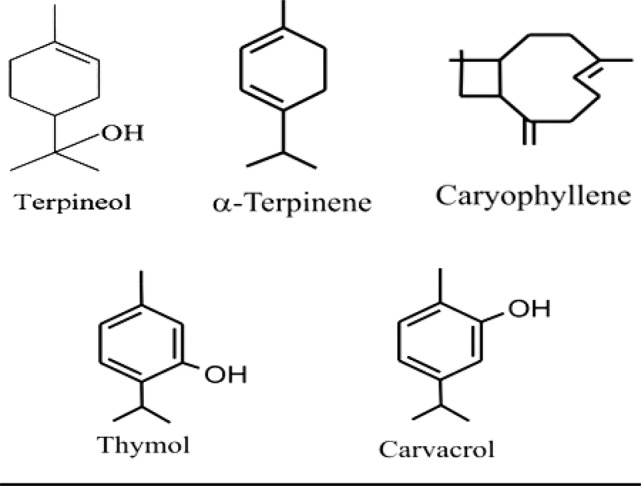
Chemical structures of major compounds of the essential oil of *Origanum vulgare*

The content of essential oil was obtained from 0.8 to 1ml per 100gr of dried leaves and the density of the essential oil was calculated as d_EO_= 0.94g mL^−1^. Oregano essential oil was found to contain 158 compounds using GC-MS. The most common compounds are shown in Table 1 and Fig. 2. In addition, chemical analysis of essential oil of *O. vulgare* is shown in Fig. 3.

**Table 1.** Constituents of oregano essential oil by GC-MS analyses

**Compound**	RI*	**Major Constituents** (%)
**Pinene**	939	1.15
**γ-Terpinene**	1056	0.14
**α – Terpinene**	1016	20.60
**α – Terpinolene**	1186	2.16
**Terpineol**	1192	22.85
**Thymol**	1290	4.53
**Carvacrol**	1299	4.9
**Caryophyllene**	1419	6.75
**Other compounds**	-	36.92

* RI, retention index as determined on an HP-5MS column using the homologous series of *n*-hydrocarbons

### Dose-response of essential oil

**Fig. 4.**
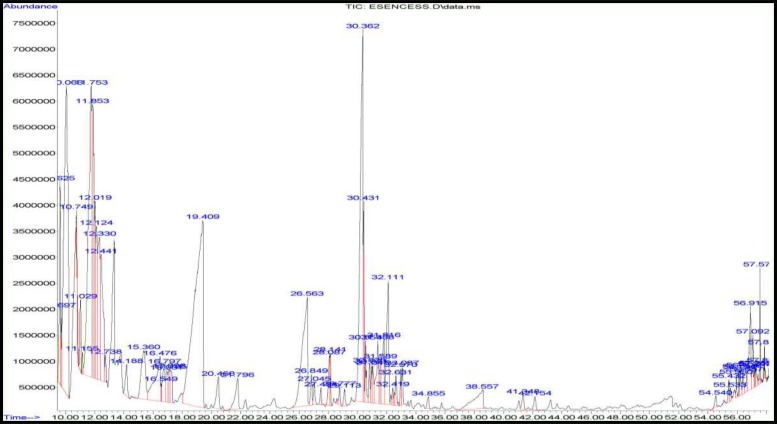
A typical GC-MS chromatogram showing the chemical analysis of essential oil from *Origanum vulgare*

The results of dose-response test are showed with the calculation of repelling effective concentration as mg essential oil per cm^2^ (EC_50_ and EC_99_) in Table 2. The dose-response data was calculated from repels effective concentration of essential oil in mg per cm^2^ of the surface. The data obtained from 9h exposure time was important because this time represents the typical time period when people are in bed and exposure to common bed bug biting. They were 1.61 and 6.57mg/cm^2^ for EC_50_ and EC_99_ at 9h after exposure, respectively with slope (±SE) = 0.469±0.073 and Chi-square (*d*f)= 17.17 and P< 0.904. Calculated dose-response curve for Oregano essential oil after 9h is shown in Fig. 4.

**Table 2.** Effective doses of Oregano essential oil against *Cimex lectularius* by treated surface method in Petri dishes

**Time**	EC_50_ (CL*) mg/cm^2^	EC_99_ (CL*) /cm^2^	**Slope (±SE)**	Chi-Square** (*df*)	**P value**
**After 3h**	0.27(0.05–0.42)	1.96 (1.52–2.97)	1.377 (±0.279)	21.57 (26)	0.712
**After 5h**	0.87(0.57–1.16)	4.10 (3.31–5.55)	0.720 (±0.112)	31.70 (26)	0.203
**After 9h**	1.61(1.18–2.09)	6.57 (5.29–8.92)	0.469 (±0.073)	17.17(26)	0.904
**After 24h**	4.53 (***)	6.10 (***)	1.19 (±0.877)	12.70 (26)	0.986

EC_50_ and EC_99_: Effective concentration cause 50% and 99% repellency against Bed Bug.

* CL: confidence limits.

** Since Chi-square goodness of fit test is not significant (P> 0.05), no heterogeneity factor is used in the calculation of confidence limits.

*** Probit model did not work because <25% repellency occurred.

### Bed bug repellency activity of essential oil

Repellency indices of oregano essential oil and 33% DEET on *C. lectularius* are shown in Table 3. The repellency means are noticeable even in very low concentrations at 3h after exposure. The Dunnett test showed, there was no significant difference in repellency between 33% DEET and oregano essential oil in the concentrations of 10, 20 and 40% (P= 0.4). After 5h, the repellent activity of oregano oil was similar to 33% DEET at the concentrations of 10% to 40% and it varied between 86% and 100%. The repellency of oregano oil at the concentrations of 10% and 20% was still obvious (86–93% repellency). Significant difference in repellency was observed between 0.625%, 1.25%, and 2.5% concentrations of oregano essential oil and 33% DEET after 3, 5 and 24h (P< 0.001), while the differences in repellency of 5%, 10%, 20%, and 40% oregano essential oil with 33% DEET were not significant after 3 and 5h (P= 0.25). The concentration of 40% oregano essential oil had 100% repellency against bed bugs at 3, 5 and 24h post exposure, while the repellency index of 33% DEET decreased to 27% after 24h (Table 3). The repellency of oregano essential oil at concentrations of 0.625–20% significantly declined from 3h to 24h, but it was constant at a concentration of 40% and exhibited similar repellency to 33% DEET at a concentration of 20%.

**Table 3.** Repellency index of essential oil, ethanol and 33% DEET against bed bugs

**Treatment**	**Concentration (%)**	**Repellency index (Mean%±SE)**			

		**3h**	**5h**	**9h**	**24h**
**Oregano essential oil**	0.625	20±0.88a	14±1.15a	13±1a	3±0.33a
	1.25	32±0.57ab	27±1.33ab	27±0.33a	7±0.57a
	2.5	44±0.88bc	39±0.33bc	34±0.88ab	10±0.33a
	5	68±0.33cd	61±0.33cd	42±0.57abc	14±0.33ab
	10	100±0.0d	86±0.33d	65±0.57bcd	17±0.57b
	20	100±0.0 d	93±0.66d	73±0.33c	25±0.57b
	40	100±0.0 d	100±0.0d	100±0.0d	100±0.0c
**33% DEET**	33	100±0.0 d	100±0.0d	100±0.0d	27±0.57b
**Ethanol**	96	-	-	-	-

Values in columns followed by different letters are significantly different (P< 0.05)

**Fig. 5.**
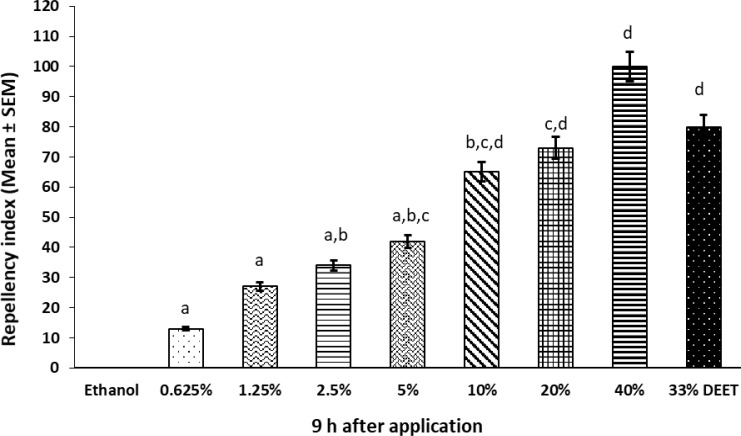
Repellency of ethanol, different concentrations of Oregano essential oil and 33% DEET against bed bugs in Petri dish assays. Different letters above the bars indicate significant differences at α= 0.05

At 9h after exposure, significant differences were observed between repellency of 0.625%, 1.25%, 2.5%, and 5% concentrations of Oregano essential oil and 33% DEET (P< 0.001), while the differences between 10%, 20%, and 40% oregano essential oil and 33% DEET were not significant (P=0.21) (Fig. 5).

## Discussion

In this study, terpineol (22.85%) was the most abundant chemical component of oregano essential oil followed by α-terpinene with 20.6% (v/v). The component of α-terpinene in *O. vulgare* collected from Mazandaran was 1.018% (38). The plant phenology and composition was likely affected by ecologic conditions. The percentage of carvacrol obtained 4.9% in the present investigation. The percentage of carvacrol, collected from various localities in West Azarbaijan Province of Iran were reported in the range of 23.54% to 67.09% (39). The chemical composition of oregano oil changed due to plants’ development, the population or variation, and the cultivation conditions. The content of carvacrol depends on climatic condition and also it is can be variable in budding period and in full bloom (40). In addition, difference in the chemical composition of oregano oil from previously published papers with our study may is regarding the type of oregano subspecies. Two percent of α-terpinene, monoterpenes derived from *Thymus vulgaris* were reported with stronger repellency activity than DEET against *Culex pipiens* (33). In our findings, oregano essential oil showed more potential as repellent compound than 33% DEET, a commercial product, against bed bugs. The high concentration α-terpinene may be responsible for the oil’s repellency.

The aim of using repellant compound towards hematophagous pest is to reduce the frequency of biting in endemic areas and prevent bites in areas where allergic reactions to biting insects are common (41). Very few studies have been conducted to evaluate insect repellent activities of essential oils against bed bugs (17, 27). More tolerance of bed bugs against insect repellents were compared with other blood-sucking insects (27).

According to the Petri dish repellency assay, the oregano essential oil of 2.5% showed 10% repellency after 24h. While the same concentration of oil demonstrated 99.1% repellent activity on the brown-banded cockroach (*Supella longipalpa*) after the same time (34). The sub-lethal concentrations (LC_10_ and LC_30_) of oregano essential oil had an average repellency of 22.8% and 49.8% against the diamondback moth (*Plutella xylustella*) respectively (42). Insect species, method of test, formulation, and concentrations of the oil were responsible for the different results observed in the reported investigations. However the repellency indices were different between selected concentrations of oregano oil and there was no significant difference between efficacy of DEET (33%) with 20% oregano oil at 3, 5, 9h post exposure. No repel activity was observed in the control group of our study and all the bed bugs stayed under harborage at 3, 5, and 9h post ethanol exposure.

Our investigation showed that repellency of 40% oregano oil was 100% against the common bed bugs in Petri dish test. It repellency index was similar to 33% DEET at 3 and 5h and it was more effective than 33% DEET at 9h after application. The 40% concentration of oregano oil was found to be safer for application in human environments and also for personal protection. While EcoRaider (1% geraniol, 1% cedar extract, and 2% sodium lauryl sulfate) and bed bug patrol (0.003% clove oil, 1% peppermint oil, and 1.3% sodium lauryl sulfate) as botanical repellents did not exhibit detectable repellency against bed bug in the presence of carbon dioxide source (17).

DEET, picaridin, tea tree oil, peppermint oil, and citronella oil were tested for repellency activity on *Triathoma rubida* using small restrained rats. No long-range repellency was observed. Only citronella oil was able to stop all probing and feeding of *T. rubida* and appears to be a promising potential repellent to prevent sleeping people from being bitten by kissing bugs (41). The efficacy of any pesticide or repellent compound can vary with the testing method, rate of application, bed bug strain and physiological stage (17).

## Conclusion

Preferably, the EC_99_ dose should be estimated to determine the protection time against blood-sucking insects. 6.57mg/cm^2^ had 99% repellency against *C. lectularius* in treated surface after 9 hours. Further studies are recommended to determine the protection time and repellency of oregano essential oil using EC_99_ on animal and human hosts.

## References

[1] Seong KM Lee DY Yoon KS Kwon DH Kim HC Klein TA Clark JM Lee SH (2010) Establishment of quantitative sequencing and filter contact vial bioassay for monitoring pyrethroid resistance in the common bed bug, *Cimex lectularius*. J Med Entomol. 47 (4): 592–599. 20695274 10.1093/jmedent/47.4.592 PMC7027264

[2] Tawatsin A Thavara U Chompoosri J Phusup Y Jonjang N Khumsawads C Bhakdeenuan P Sawanpanyalert P Asavadachanukorn P Mulla MS (2011) Insecticide resistance in bedbugs in Thailand and laboratory evaluation of insecticides for the control of *Cimex* *hemipterus* and *Cimex lectularius* (Hemiptera: Cimicidae). J Med Entomol. 48: 1023–1030. 21936321 10.1603/me11003

[3] Politi FAS Nascimento JD da Silva AA Moro IJ Garcia ML Guido RVC Pietro RCL Godinho AF Furlan M (2016) Insecticidal activity of an essential oil of *Tagetes patula* L. (Asteraceae) on common bed bug *Cimex lectularius* L. and molecular docking of major compounds at the catalytic site of ClAChE1. Parasitol Res. 116(1): 415–424. 27838836 10.1007/s00436-016-5305-x

[4] Haghi SFM Behbodi M Hajati H Shafaroudi MM (2014) Prevalence of bed bug (*Cimex* *lectularius*) in human settlement area of Bahnamir, Iran. Asian Pac J Trop Dis. 4: S786–S789.

[5] Giorda F Guardone L Mancini M Accorsi A Macchioni F Mignone W (2013) Cases of bed bug (*Cimex* *lectularius*) infestations in Northwest Italy. Vet Ital. 49(4): 335–340. 24362773 10.12834/VetIt.1011.10

[6] How YF Lee CY (2010) Survey of bed bugs in infested premises in Malaysia and Singapore. J Vector Ecol. 35(1): 89–94. 20618653 10.1111/j.1948-7134.2010.00033.x

[7] Omudu E Kuse C (2010) Bedbug infestation and its control practices in Gbajimba: a rural settlement in Benue state, Nigeria. J Vector Borne Dis. 47(4): 222–227. 21178215

[8] Levy Bencheton A Berenger J Del Giudice P Delaunay P Morand J (2011) Resurgence of bedbugs in southern France: a local problem or the tip of the iceberg? JEADV. 25(5): 599–602. 20704629 10.1111/j.1468-3083.2010.03804.x

[9] El-Azazy O Al-Behbehani B Abdou N (2013) Increasing bedbug, *Cimex lectularius*, infestations in Kuwait. J Egypt Soc Parasitol. 43(2): 415–418. 24260819 10.12816/0006397

[10] Doggett SL Russell RC (2008) The resurgence of bed bugs, *Cimex* spp. (Hemiptera: Cimicidae) in Australia. The 6th International Conference on Urban Pests, 2008 Veszprem: OOK-Press Kft.

[11] Kilpinen O Jensen KMV Kristensen M (2008) Bed bug problems in Denmark, with a European perspective. The 6th International Conference on Urban Pests, 2008 OOK-Press Veszprém pp. 13–16.

[12] Hwang SW Svoboda TJ De Jong IJ Kabasele KJ Gogosis E (2005) Bed bug infestations in an urban environment. Emerg Infect Dis. 11(4): 533. 15829190 10.3201/eid1104.041126 PMC3320350

[13] Potter MF (2006) The perfect storm: an extension view on bed bugs. Am Entomol. 52(2): 102–104.

[14] Reinhardt K Harder A Holland S Hooper J Leake-Lyall C (2008) Who knows the bed bug? Knowledge of adult bed bug appearance increases with people’s age in three counties of Great Britain. J Med Entomol. 45(5): 956–958. 18826041 10.1603/0022-2585(2008)45[956:wktbbk]2.0.co;2

[15] Goddard J de Shazo R (2012) Psychological effects of bed bug attacks (*Cimex lectularius* L.). Am J Med. 125(1): 101–103. 22195533 10.1016/j.amjmed.2011.08.010

[16] Ab Majid AH Zahran Z (2015) Laboratory bioassay on efficacy of dual mode of action insecticides (beta-cyfluthrin and imidacloprid) towards tropical bed bugs, *Cimex hemipterus* (Hemiptera: Cimicidae). J Entomol Zool Stud. 3(5): 217–220.

[17] Singh N Wang C Cooper R (2014) Potential of essential oil-based pesticides and detergents for bed bug control. J Econ Entomol. 107(6): 2163–2170. 26470082 10.1603/EC14328

[18] Romero A Potter MF Potter DA Haynes KF (2007) Insecticide resistance in the bed bug: a factor in the pest’s sudden resurgence? J Med Entomol. 44(2): 175–178. 17427684 10.1603/0022-2585(2007)44[175:IRITBB]2.0.CO;2

[19] Debboun M Strickman DA Klun JA (2005) Repellents and the military: our first line of defense. J Am Mosq Control Assoc. 21(Supplement 1): 4–6. 16921676 10.2987/8756-971X(2005)21[4:RATMOF]2.0.CO;2

[20] McCabe E Barthel W Gertler S Hall S (1954) Insect Repellents. Iii. N, N-Diethylamides1. J Org Chem. 19(4): 493–498.

[21] Yaghoobi-Ershadi M Akhavan A Jahanifard E Vatandoost H Amin G Moosavi L Ramazani AZ Abdoli H Arandian M (2006) Repellency effect of Myrtle essential oil and DEET against *Phlebotomus papatasi*, under Laboratory Conditions. Iran J Public Health. 35(3): 7–13.

[22] McGready R Hamilton KA Simpson JA Cho T Luxemburger C Edwards R Looareesuwan S White NJ Nosten F Lindsay SW (2001) Safety of the insect repellent N, N-diethyl-M-toluamide (DEET) in pregnancy. Am J Trop Med Hyg. 65(4): 285–289. 11693870 10.4269/ajtmh.2001.65.285

[23] Goodyer L Behrens RH (1998) Short report: the safety and toxicity of insect repellents. Am J Trop Med Hyg. 59 (2): 323–324. 9715955 10.4269/ajtmh.1998.59.323

[24] Tripathi AK Upadhyay S Bhuiyan M Bhattacharya P (2009) A review on prospects of essential oils as biopesticide in insect pest management. J Pharmacogn Phytochem. 1(5): 052–063.

[25] Maia MF Moore SJ (2011) Plant-based insect repellents: a review of their efficacy, development and testing. Malar J. 10(1): S11. 21411012 10.1186/1475-2875-10-S1-S11 PMC3059459

[26] Koul O Walia S Dhaliwal G (2008) Essential oils as green pesticides: potential and constraints. Biopestic Int. 4(1): 63–84.

[27] Wang C Lü L Zhang A Liu C (2013) Repellency of selected chemicals against the bed bug (Hemiptera: Cimicidae). J Econ Entomol. 106(6): 2522–2529. 24498754 10.1603/ec13155

[28] Nerio LS Olivero-Verbel J Stashenko E (2010) Repellent activity of essential oils: a review. Bioresour Technol. 101 (1): 372–378. 19729299 10.1016/j.biortech.2009.07.048

[29] Khater HF (2012) Prospects of botanical biopesticides in insect pest management. Pharmacol. 3(12): 641–656.

[30] Kayedi MH Haghdoost AA Salehnia A Khamisabadi K (2014) Evaluation of Repellency Effect of Essential Oils of *Satureja khuzestanica* (Carvacrol), *Myrtus communis* (Myrtle), *Lavendula officinalis* and *Salvia sclarea* using Standard WHO Repellency Tests. J Arthropod Borne Dis. 8(1): 60–68. 25629066 PMC4289512

[31] Mohan M Haider SZ Andola HC Purohit V (2011) Essential oils as green pesticides: for sustainable agriculture. Res J Pharm Biol Chem Scie. 2(4): 10–104.

[32] Park BS Choi WS Kim JH Kim KH Lee SE (2005) Monoterpenes from Thyme (*Thymus vulgaris*) as potential mosquito repellents. J Am Mosq Control Assoc. 21(1): 80–3. 15825766 10.2987/8756-971X(2005)21[80:MFTTVA]2.0.CO;2

[33] Choi WS Park BS Ku SK Lee SE (2002) Repellent activities of essential oils and monoterpenes against *Culex pipiens pallens*. J Am Mosq Control Assoc. 18(4): 348–351. 12542193

[34] Sharififard M Safdari F Siahpoush A Kassiri H (2016) Evaluation of Some Plant Essential Oils against the Brown-Banded Cockroach, *Supella* *longipalpa* (Blattaria: Ectobiidae): A Mechanical Vector of Human Pathogens. J Arthropod Borne Dis. 10(4): 528–537. 28032105 PMC5186743

[35] Dewer Y Mahmoud MM (2014) Effectiveness and safety of some essential oils of aromatic plants on the growth and silk production of the silkworm *Bombyx mori* L. J Entomol Zool Stud. 2(2): 81–86.

[36] Adams RP (2007) Identification of essential oils by gas chromatography/mass spectrometry. Carol Stream, Allured Publishing Corporation.

[37] Robertson JL Savin N Preisler HK Russell RM (2007) Bioassays with arthropods, CRC press.

[38] Hashemi M Ehsani A Aminzare M Hassanzadazar H (2016) Antioxidant and Antifungal Activities of Essential Oils of *Origanum vulgare* ssp. gracile Flowers and Leaves from Iran. J food qual hazards control. 3(4): 134–140.

[39] Pirigharnaei M Zare S Heidary R Khara J EmamaliSabzi R Kheiry F (2011) The essential oils compositions of Iranian Oregano (*Origanum vulgare* L.) populations in field and provenance from Piranshahr District, West Azarbaijan Province, Iran. Avicenna J Phytomed. 1(2): 106–114.

[40] Nurzynska-Wierdak R (2009) Herb yield and chemical composition of common oregano (*Origanum* *vulgare* L.) essential oil according to the plant’s developmental stage. Herba polonica 55(3): 55–62.

[41] Terriquez J Klotz SA Meister E Klotz J Schmidt J (2013) Repellency of DEET, picaridin, and three essential oils to *Triatoma rubida* (Hemiptera: Reduviidae: Triatominae). J Med Entomol. 50(3): 664–667. 23802464 10.1603/me12282

[42] Nasr M Sendi JJ Moharramipour S Zibaee A (2015) Evaluation of *Origanum vulgare* L. essential oil as a source of toxicant and an inhibitor of physiological parameters in diamondback moth, *Plutella xylustella* L. (Lepidoptera: Pyralidae). J Saudi Soci Agric Sci. 16: 184–190.

